# Impact of miRNA Sequence on miRNA Expression and Correlation between miRNA Expression and Cell Cycle Regulation in Breast Cancer Cells

**DOI:** 10.1371/journal.pone.0095205

**Published:** 2014-04-18

**Authors:** Zijun Luo, Yi Zhao, Robert Azencott

**Affiliations:** 1 Harbin Institute of Technology, Shenzhen Graduate School, Shenzhen, China; 2 University of Houston, Houston, Texas, United States of America; Rutgers - New Jersey Medical School, United States of America

## Abstract

The miRNAs regulate cell functions by inhibiting expression of proteins. Research on miRNAs had usually focused on identifying targets by base pairing between miRNAs and their targets. Instead of identifying targets, this paper proposed an innovative approach, namely impact significance analysis, to study the correlation between mature sequence, expression across patient samples or time and global function on cell cycle signaling of miRNAs. With three distinct types of data: The Cancer Genome Atlas miRNA expression data for 354 human breast cancer specimens, microarray of 266 miRNAs in mouse Embryonic Stem cells (ESCs), and Reverse Phase Protein Array (RPPA) transfected by 776 miRNAs in MDA-MB-231 cell line, we linked the expression and function of miRNAs by their mature sequence and discovered systematically that the similarity of miRNA expression enhances the similarity of miRNA function, which indicates the miRNA expression can be used as a supplementary factor to predict miRNA function. The results also show that both seed region and 3' portion are associated with miRNA expression levels across human breast cancer specimens and in ESCs; miRNAs with similar seed tend to have similar 3' portion. And we discussed that the impact of 3' portion, including nucleotides 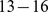, is not significant for miRNA function. These results provide novel insights to understand the correlation between miRNA sequence, expression and function. They can be applied to improve the prediction algorithm and the impact significance analysis can also be implemented to similar analysis for other small RNAs such as siRNAs.

## Introduction

The miRNAs are small non-coding RNAs of roughly 22 nucleotides in length, which can bind with and inhibit protein coding mRNAs through complementary base pairing. A given miRNA can potentially bind and silence hundreds of mRNAs across a number of signaling pathways. By degrading mRNAs and repressing proteins, miRNAs regulate the cell signaling and cell functions.

On the correlation between sequence and function of miRNAs, the central goal of the past research had been to understand how they recognize their target messages. The best characterized features determining the targets of a specific miRNA are the conserved Watson-Crick pairing to the seed (positions 2–7) of the miRNA, which is the so-called "seed pairing rules". [1]–[5]. Seed rules have been informative [6] to predict targets of miRNAs, especially in combination with microarray or proteomic approaches [7]–[9].

However, seed pairing rules do not always confer repression of target messages. Efforts have been made to explain that perfect complementarity between miRNA seed and mRNA 3' UTR are neither necessary nor sufficient for all functional miRNA-target interactions [10]–[14]. False predictions from seed rules could be explained as target transcripts with non-canonical target sites. Chi et al. proposed an alternative mode of miRNA target prediction [14] and identified functional non-canonical miRNA-mRNA interactions.

To improve the accuracy of predicting miRNA targets, Grimson et al. proposed determinants for targeting beyond seed pairing [12]. The 3' portion of miRNA mature sequence had been identified as one of the additional "context" features that correlate with reduced expression levels of mRNAs. Many target prediction algorithms, including TargetScan and miRanda [2], [5], [15]–[17], use base pairing of the 3' portion as an important factor to predict targets though the correlation between 3' portion and targeting is weak.

Besides identification of gene/protein downregulation induced by overly expressed miRNAs, classical microarray analysis also relies on massive application of linear correlation analysis to gene/protein expression profiles [18]. Nonlinear chemical kinetics modeling approach were also proposed to numerically validate potential miRNA-mRNA pairs [19]. Either linear or nonlinear approach implicitly classifies miRNAs with similar time-course expression levels as candidates to have similar regulatory roles. However, it has not been systematically analyzed whether there is a significant correlation between the expression levels and the regulatory function of miRNAs.

In this paper, we studied the correlation between sequence, expression and function of miRNAs by a novel method based on three different sets of data. Interestingly, similar results were obtained by the same analysis to the TCGA miRNA expression data for 354 breast cancer specimens, miRNA time-course expression data in ESCs and the RPPA transfected by miRNAs in MDA-MB-231 cell line. Our analysis focused only on the "input" (mature sequence) and "output" (miRNA expression and function) without regard to target sites. We linked the expression of miRNAs with their cell cycle regulation and found that miRNA expression (across patient samples or across time) can be considered as a supplementary factor to predict miRNA function. The seed region influence both miRNA expression (across patient samples or across time) and cell cycle signaling independently while the 3' portion is significantly effective for miRNA expression but not for cell cycle regulation. Although the 3' portion had been claimed to have impact on miRNA function[12], we discussed why the 3' portion was rarely found effective. The results provide novel insights to understand correlation between miRNA sequence, expression and function in cells. They can also be applied to improve target prediction algorithms.

## Results

### The Impact of the Seed and the 3' Portion on miRNA Expression and Cell Cycle Regulation

For the expression data of 321 miRNAs across 354 breast cancer specimens, we had calculated the expression distance for each of 
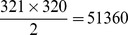
 miRNA pairs.

**Figure 1:**
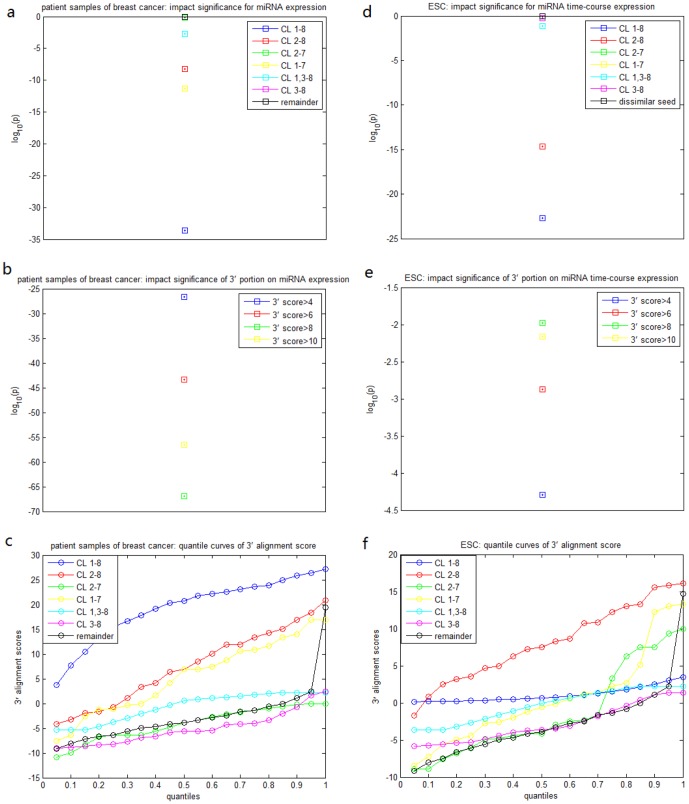
IS analysis for miRNA expression data. a. impact significance (IS) of nucleotides 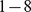, 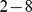, 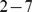, 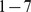, 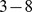, and 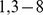 for TCGA miRNA expression across breast cancer specimens after excluding miRNA pairs with high alignment score (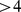) of the 3' portion. b. IS of the 3' portion for TCGA expression after excluding miRNA pairs with identical nucleotides 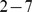 or 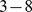. Threshold of similar 3' portion is set to be 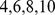 respectively. c. quantile curves of 3'-alignment scores for 7 groups from the TCGA expression data: the 6 classes and the remaining pairs. Groups with quantile curves above tend to be more similar in the 3' portion than groups with quantile curves below. KS test shows that CL 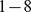 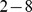, and 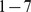 tend to have more similar 3' portion (p values: 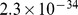, 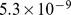 and 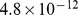) d. e. f. same analysis as a. b. c. for miRNA time-course expression in ESCs.

We first studied the impact significance of the seed region (nucleotides 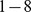) and the 3' portion separately. The set of all miRNA pairs was divided into the following 6 disjoint classes: nucleotides 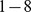 identical (CL 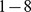); nucleotides 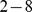 identical, nucleotide 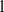 different (CL 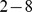); nucleotides 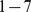 identical, nucleotide 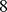 different (CL 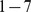); nucleotides 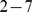 identical, nucleotides 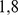 different (CL 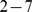); nucleotides 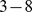 identical, nucleotides 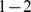 different (CL 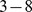); nucleotides 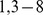 identical, nucleotide 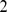 different (CL 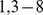). Excluding the miRNA pairs with an alignment score of the 3' portion larger than a threshold of 4, we calculated the impact significance of the 6 classes. When we changed the threshold to 6 and to 8, the IS values stay almost the same. Figure 1a shows that CL 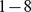, CL 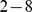, and CL 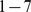 are significantly associated with miRNA expression. The association of CL 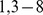 is weak (p value = 0.0018) while CL 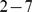 and CL 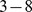 have no significant impact.

Similarly, we calculated the IS values of the 3' portion for the miRNA pairs excluding those with identical nucleotides 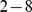. Varying thresholds for "high" alignment of the 3' portion (alignment score 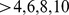), we can see (Figure 1b) that the 3' portion has a significant influence on miRNA expression.

**Figure 2:**
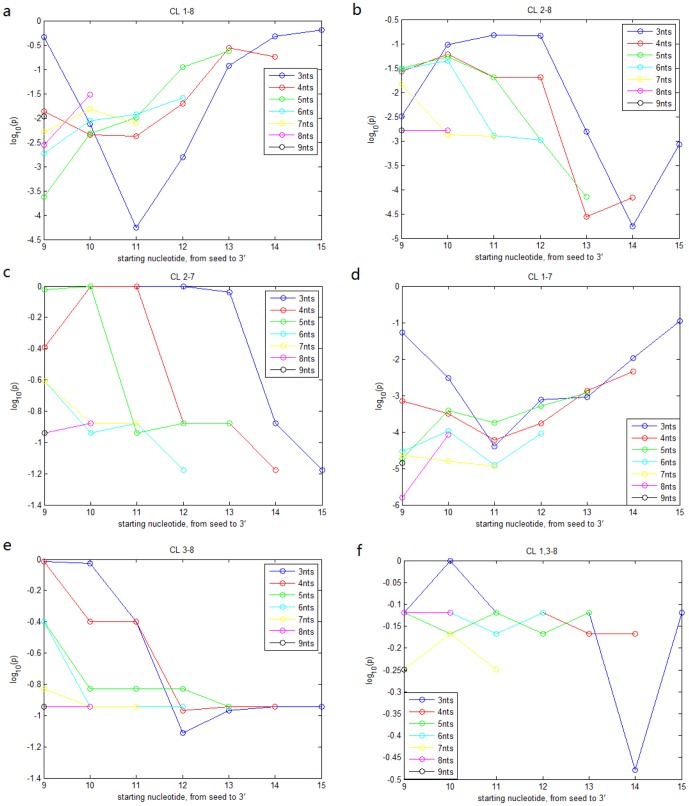
IS values of the searched contiguous segments of the 3' portion for each of the 6 classes from the TCGA expression data. 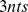
 represents 3 contiguous nucleotides, 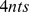 4 contiguous nucleotides, and so forth. The x-axis represents the starting position of the segments.

To examine whether the 3' portion influences miRNA expression when the seeds are identical or highly similar, we searched the impact significance of all possible contiguous segments (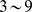 nt) in the 3' portion in each of the six classes. It turned out that segments 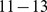, 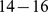 and 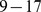 are mostly influential (with smallest p values) for CL 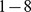, CL 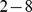 and CL 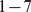 respectively (Figure 2). And the 3' portion is not effective for CL 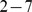, CL 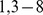 and CL 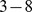 at all. Although the 3' portion dissociates with the seed region to impact miRNA expression, we examined the correlation between the alignment scores of the seed region and the 3' portion and found that higher similarity in the seed region tends to be accompanied with higher similarity in the 3' portion (Figure 1c). Classes CL 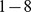, CL 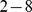 and CL 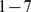 tend to be more similar in the 3' portion than CL 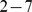, CL 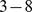, CL 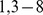 and the remaining pairs (Figure 1c).

**Figure 3:**
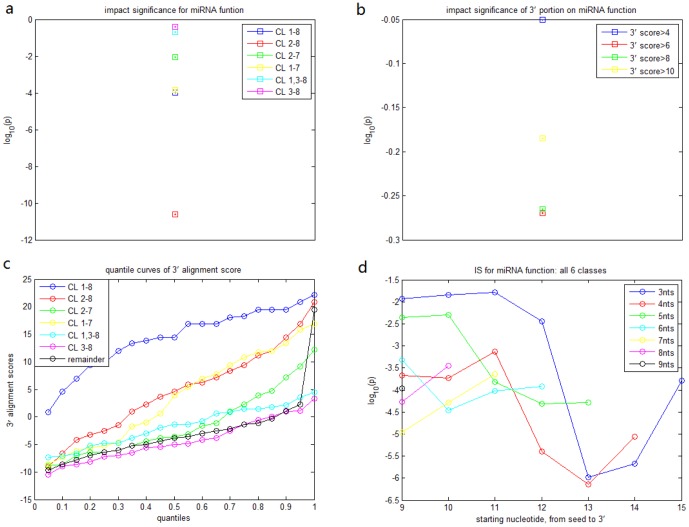
IS analysis for RPPA data. a. IS of nucleotides 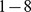, 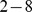, 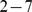, 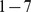, 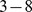, and 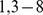 for miRNA cell cycle regulation in breast cancer cells after excluding miRNA pairs with high alignment score (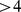) of the 3' portion. b. IS of the 3' portion for miRNA cell cycle regulation in breast cancer cells after excluding miRNA pairs with identical nucleotides 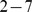 or 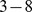. Threshold of similar 3' portion is set to be 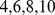 respectively. c. quantile curves of 3'-alignment scores for 7 groups from the breast cancer RPPA data: CL 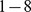, 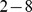, 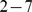, 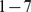, 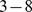, 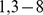 and the remaining pairs. Groups with quantile curves above tend to be more similar in the 3' portion than groups with quantile curves below. KS test shows that CL 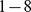, 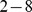, 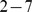 and 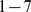 tend to have more similar 3' portion (p values: 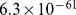, 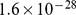, 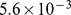, 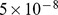) d. IS values of the searched contiguous segments of the 3' portion for all 6 classes from the breast cancer RPPA data. 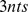 represents 3 contiguous nucleotides, 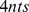 4 contiguous nucleotides, and so forth. The x-axis represents the starting position of the segments.

**Figure 4:**
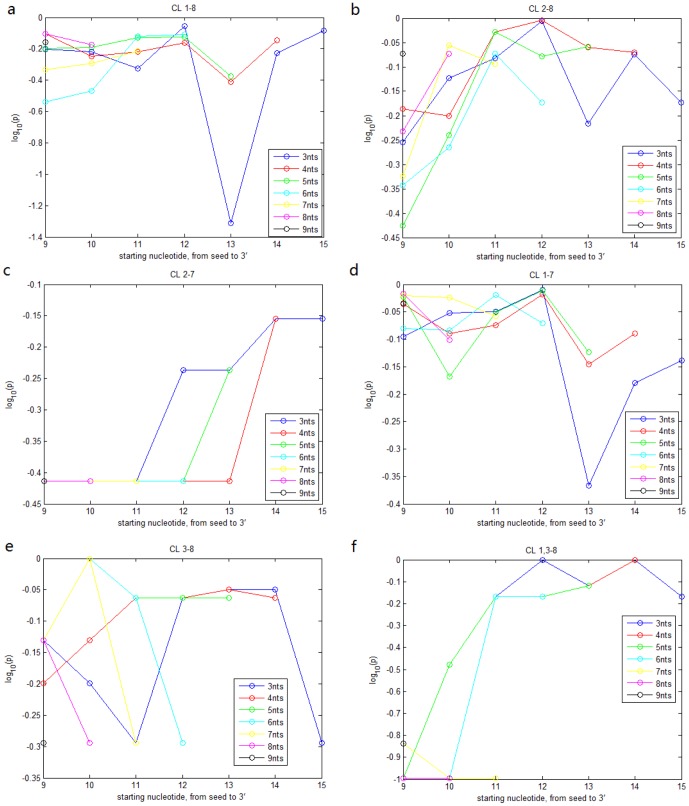
IS values of the searched contiguous segments of the 3' portion for each of the 6 classes from the expression data of ESCs. 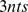
 represents 3 contiguous nucleotides, 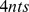 4 contiguous nucleotides, and so forth. The x-axis represents the starting position of the segments.

**Figure 5:**
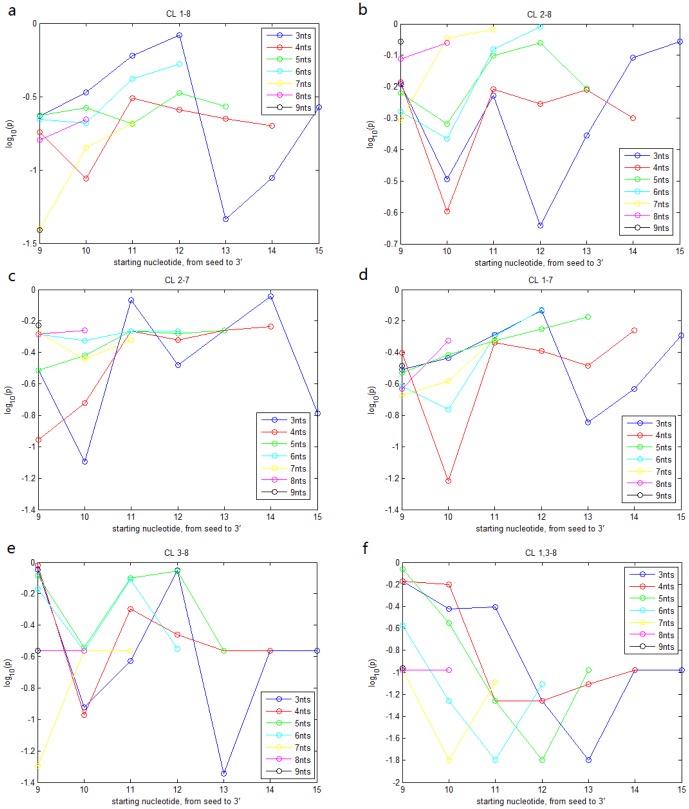
IS values of the searched contiguous segments of the 3' portion for each of the 6 classes from the RPPA data. 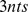
 represents 3 contiguous nucleotides, 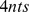 4 contiguous nucleotides, and so forth. The x-axis represents the starting position of the segments.

We applied the same analysis to the miRNA time-course expression data in ESCs and the RPPA transfected by 776 miRNAs in MDA-MB-231 cell line and obtained quite similar results (Figures 1d, 1e, 1f, 3a, 3b, 3c). Compared with the results for miRNA expression across TCGA breast cancer specimens, CL 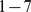 is not significantly associated with miRNA time-course expression in ESCs. Figure 4 also indicate that the 3' portion is not influential when the seed is highly similar for miRNA time-course expression in ESCs. Figures 3b and 5 show that the impact of the 3' portion is not significant for miRNA function in cell cycle signaling in contrast to its impact on miRNA expression.

### Similarity of miRNA Expression Enhances Similarity of miRNA Cell Cycle Regulation

**Figure 6:**
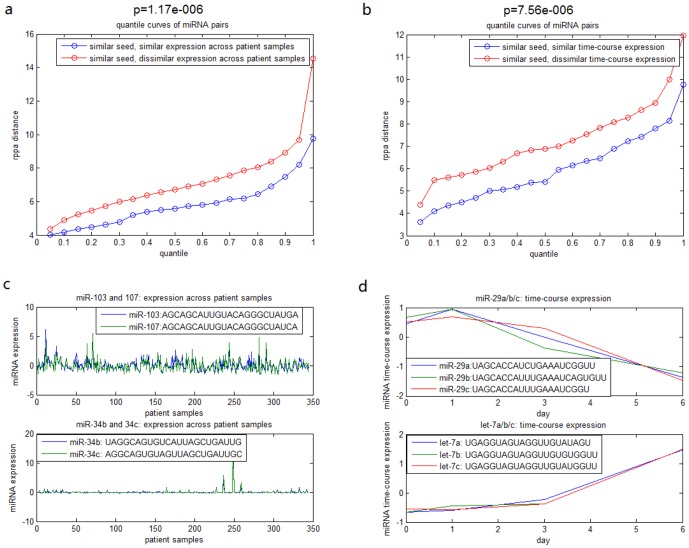
Correlation between miRNA expression and function. a. quantile curves of two groups for TCGA expression data. group 1: miRNA pairs of similar seed (seed alignment score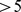) and similar expression (expression distance 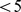), group 2: miRNA pairs of similar seed (seed alignment score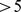) and dissimilar expression distance (expression distance 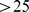). KS test shows that group 1 tend to have more similar cell cycle regulation than group 2 (p value: 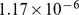). b. quantile curves of two groups for expression data of ESCs. group 1: miRNA pairs of similar seed (seed alignment score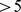) and similar expression (expression distance 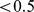), group 2: miRNA pairs of similar seed (seed alignment score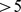) and dissimilar expression distance (expression distance 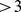). KS test shows that group 1 tend to have more similar cell cycle regulation than group 2 (p value: 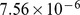). c. expression levels across breast cancer specimens and mature sequences of two miRNA pairs. d. time-course expression levels of ESCs and mature sequences of two miRNA pairs.

Based on the above results, it is natural to expect similarity of miRNA expression might be useful to predict similarity of miRNA function. Since the seed, but not the 3' portion, is significantly effective for breast cancer cell regulation, we studied only miRNA pairs with similar seed region and found that indeed similar expression across TCGA breast cancer specimens enhances similarity of cell cycle regulation(Figures 6a). Even when these miRNAs are from different cells (Figure 6b), our statistical analysis still proved that there is a strong correlation between miRNA time-course expression in ESCs and cell cycle regulation in breast cancer cells. Actually, several tested examples of different cell types also support the result, which we discuss in the section below. Therefore, miRNAs with similar seed and expression (across samples) are good candidates to have similar regulatory roles, in other words, miRNA expression (across samples) can be considered as a supplementary determinant to predict the miRNA function.

In conclusion, nucleotides 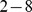 and 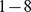 are influential on both miRNA expression/function while the 3' portion is significantly effective only for expression of miRNAs; the 3' portions tend to be similar when the seed regions are similar; miRNAs with similar mature seed and expression (across samples) are good candidates to have similar regulatory roles.

## Discussion

To analyze the correlation between mature sequence, expression and global function of miRNAs, we defined IS by computing the p-values of Kolmogorov Smirnov tests applied to compare Euclidean distances of observations of miRNA pairs. This technique allows us to study the differences of global function for distinct miRNAs and the influence of sequence on cell signaling without identifying the targets of these miRNAs. We hence avoided the complexity to determine canonical or noncanonical target sites of UTR and focused on only the miRNA sequence as input and the regulation effect (RPPA) or expression as output. Another advantage of our similarity analysis is that it could be used for clustering miRNAs. For those miRNAs with highly similar seed and expression, it is reasonable to hypothesize that the miRNA cluster have similar regulatory function.

### Literature Support for Association between miRNA Expression and Function

In this paper, we also systematically linked the miRNA expression with their function by alignment score of miRNA sequence. There are some tested examples to support the association between miRNA function, mature sequence, and expression. For instance, miR-103 and miR-107, having very similar mature sequence and expression levels (Figure 6c), are two known miRNAs that have the same roles in regulating insulin sensitivity and promoting metastasis of colorectal cancer [20], [21]; miR-34b and miR-34c, having very similar mature sequence and expression levels (Figure 6c), are targets of p53 and cooperate in control of cell proliferation and adhesion-independent growth[22]; let-7a/b/c were also claimed to reduces tumor growth in mouse models of lung cancer [23] and miR-29a/b/c reverts aberrant methylation in lung cancer by targeting DNA methyltransferases 3A and 3B [24] while these two miRNA clusters have very similar mature sequences and expression levels (Figure 6d). Thus, we may infer that the conclusion of the paper also holds for expression data and RPPA transfected by miRNAs in different types of cells.

### Noneffectiveness of the 3' Portion, Especially Segment 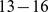 for miRNA Cell Cycle Regulation

If we consider the impact significance of CL 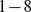 is corresponding to the downregulation of 8mer match of seed region; CL 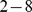 to 7mer-m8 match; CL 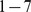 to 7mer-A1 match; CL 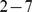 to 6mer match; then our results about the impact of the seed region for miRNA function are quite consistent with those in [12].

Although Grimson et al. [12] revealed that the 3' portion, especially nucleotides 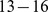, enhance the repression of canonical 7mer or mismatched seed sites, our results indicate that the impact of 3' portion is not significant for miRNA function. Systematic examination of site conservation indicates that mismatched seed sites with 3'-compensatory pairing are only rarely under selective pressure to be conserved [5]. So the statistical difference of results between [12] and our paper could be well explained by the difference in size of the studied data (only 11 miRNAs and microarray data were studied in [12], while RPPA transfected by 776 miRNAs in this paper). To explore deeper the impact of the 3' portion, we examined the miRNA pairs with identical nucleotides 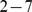 or 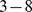 (including all 6 classes) and found that nucleotides 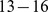 seems to most significantly impact the miRNA function (Figures 3d), which is quite consistent with the results of [12].

The above about the impact of 3' portion seems contradictory to our previous results (Figure 5). In fact, CL 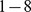, 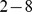 and 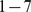, having more similar 3' portion (Figure 3c), are more strongly associated with similar miRNA function than CL 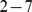, 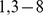 and 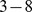. Consequently the IS analysis shows that the 3' portion (especially nucleotides 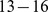) is significantly influential among all miRNA pairs in our 6 classes. Therefore, the 3' portion seems to be effective because it has a strong association with the seed region, which apparently impacts the miRNA function.

Since our results show that the 3' portion is not effective when the seeds are highly similar, it may be unreasonable to assume that the seed and the 3' region have synergistic influence for the prediction of targets. This remark applies to miRanda algorithm for miRNA target prediction that uses a weighted sum of match and mismatch scores for base pairs, which is a linear model and implies that the seed and 3' region act synergistically.

In summary, our results provide novel insights on the impact and correlation between the seed, the 3' portion, miRNA expression and function. It is proposed that miRNA expression (across samples) can be used as a supplementary factor to predict the miRNA function. These results are also useful to improve target prediction models and algorithms. Our approach can handle large proteomic or genomic data easily and study the global function of miRNAs instead of their targets. It is a novel and easy to use technique to study miRNA expression/function and it could also be possibly applied to similar analysis for other small RNAs such as siRNAs.

## Materials and Methods

### Data Description

The expression profiles of miRNAs across 354 patient samples of breast invasive carcinoma are publicly available on The Cancer Genome Atlas (TCGA: https://tcga-data.nci.nih.gov/tcga/dataAccessMatrix.htm). Level 3 miRNA expression data for 354 breast cancer specimens profiled using Illumina GAIIx were downloaded from the TCGA data portal (select a disease: breast invasive carcinoma, data type: miRNASeq, data level: level 3). Mature sequences of 321 individual miRNAs in the list of the paper were collected from miRBase (www.mirbase.org).

The microarray data of mouse ESCs undergoing RA-induced differentiation, are also publicly accessible from the paper by Gu et al. [18] (see table S1 for the normalized data). The miRNAs microarray was provided by LC Science Inc. Expression levels were recorded for 266 well characterized miRNAs on days 0, 1, 3, 6, based on 8 probe replicates for miRNAs (mmu-miRs).

The RPPA data transfected by miRNAs are publicly accessible online from in the paper by Uhlmann et al. 2011 [25]. Mature sequences of 776 individual miRNA mimics used in our paper were collected from miRBase. Normalized signal intensities of 26 proteins in cell cycle pathway were recorded in each well transfected by miRNAs in MDA-MB-231 cell line (see table S2 for the normalized RPPA data collected from table S5 in [25] and the mature sequence from miRBase).

### Data Pretreatment

For the TCGA expression data, we eliminated all miRNA profiles with extremely low expression (less than 20) in all the samples and thus obtained the profiles of 321 distinct miRNAs. The expression levels across 354 patients for each miRNA had been normalized by its own mean and standard deviation. For the time-course data of miRNAs, the expression levels of each day were averaged for replicates. After taking logarithm of the data, each observation of the time-course expression levels of the 266 miRNAs were normalized by its own mean value and standard deviation.

### Methods

We first defined the RPPA distance of any miRNA pair (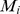, 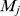) to be the Euclidean distance of the RPPA data 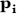 and 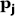, where 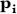 is the vector 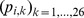 and 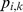 represents the normalized expression of 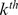 protein transfected by miRNA 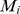. Similarly, we defined the expression distance of any miRNA pair (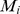, 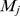) to be the Euclidean distance of the expression data 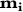 and 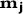, where 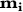 is the vector 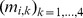 and 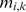 represents the normalized expression at 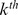 patient samples or time point of miRNA 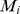.

The alignment score between any miRNA pair (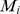, 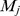) were computed by Needleman-Wunsch algorithm (Matlab function: nwalign). Let 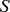 be a subsequence of miRNA mature sequence, such as for instance nucleotides 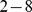. For any subsequence 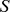, we quantify the similarity of two miRNAs by their alignment score 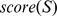 on sequence 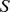. Then we can divide all the available miRNA pairs (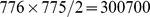 pairs for the RPPA data, 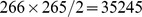 for the miRNA time-course data) into two groups: let group 1 be the group of miRNA pairs with high alignment 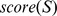, group 2 be the group of miRNA pairs with low alignment 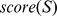. We used Kolmogorov-Smirnov (KS) test to compare the distribution of rppa/expression distance for the two groups and got a p value 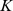. We define "impact significance" (IS) of 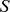, where 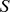 denotes a given segment of the miRNA mature sequence, by the formula:
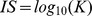
Note that if 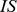 is smaller than some threshold such as 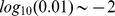, then it implies that for miRNAs pairs with strong 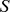 similarity, the rppa/expression tend to be similar.

Our approach focused on the similarity of miRNA pairs in sequence, expression and global function, while traditional methods focused on base-pairing between miRNA and target messages, and downregulation effects.

## Supporting Information

Table S1 **miRNA mature sequence and normalized expression at day 0, 1, 3, 6 in mouse ES cells.**
(XLS)

Table S2 **miRNA sequence and RPPA data of MDA-MB-231 cell line transfected by each individual miRNA.**
(XLS)
